# Penetrating Neck Injury in a Child Presenting as a Simple Laceration: A Case Report

**DOI:** 10.5811/cpcem.52830

**Published:** 2026-06-26

**Authors:** Brian Davitt, Eric Quinn

**Affiliations:** * Maimonides Medical Center, Department of Pediatric Emergency Medicine, Brooklyn, New York; † Geisinger Health System, Department of Emergency Medicine, Danville, Pennsylvania

**Keywords:** penetrating neck injury, pediatric, laceration, glass foreign body, case report

## Abstract

**Introduction:**

Penetrating neck injury is rare in children, but it can have devastating consequences due to the vital structures in the neck. Having a high index of suspicion is crucial for detection and management of such injuries. Superficial-appearing wounds can mask underlying injury. In this case report, we present a case of penetrating neck injury in a patient who presented with what appeared to be a simple laceration to his neck.

**Case Report:**

A three-year-old male presented with a laceration to his neck caused by a piece of shattered glass. On evaluation, he had normal vital signs and no hard or soft signs of penetrating neck injury. During bedside examination, a track was found extending beyond the platysma. The patient developed hoarseness in his voice, prompting computed tomography (CT). The CT revealed three foreign bodies lodged between the right common carotid artery and tracheal wall, abutting the wall. The patient underwent open exploration with removal of the foreign bodies. No significant damage to vital structures was found.

**Conclusion:**

While rare, this case exemplifies the importance of having a high index of suspicion for penetrating neck injury in children presenting with even superficial-appearing neck lacerations, especially if caused by glass. Because the neck is typically uncovered, it carries an increased risk for foreign body penetration. Glass is generally radiopaque, but detection may be limited by the surrounding tissue, fragment size, and glass composition. Patients demonstrating hard signs of penetrating neck injury require operative management, while those with soft signs should undergo assessment with CT.

## INTRODUCTION

Lacerations are a common reason for children presenting to the emergency department (ED). Injuries involving the neck warrant vigilance, as penetrating trauma may be mistakenly regarded as a superficial laceration during initial evaluation. We present a case of a three-year-old healthy male who presented to the pediatric ED with a laceration to the neck. The injury appeared to be a simple laceration, with no active bleeding. Eventually, however, he developed a soft sign of a penetrating neck injury, leading to computed tomography (CT), which showed multiple foreign bodies adjacent to vital structures, requiring wound exploration and foreign body removal in the operating room.

This case is notable because the patient presented with what initially appeared to be a simple laceration. Without further evaluation, the wound might have been repaired and the patient discharged, risking complications including injury to vital cervical structures, delayed wound healing, deep neck space infection, fistula formation, or foreign body migration.^1^ It illustrates the necessity of considering penetrating neck injury in a child presenting with neck lacerations and underscores the importance of considering the mechanism of injury in guiding evaluation.

## CASE REPORT

A three-year-old male with no significant past medical history presented to the pediatric ED with a laceration to the neck. According to the parents’ interpretation of the event, the patient had reached up into a cabinet to grab a glass cup. The cup shattered, and a piece hit his anterior neck, resulting in a laceration. He also sustained a laceration to the second left digit when the cup broke. No other injuries were noted by the parents, and there was no active bleeding. The patient had no difficulty breathing, was able to phonate, and had normal age-appropriate vital signs in triage.

**Image 1:**
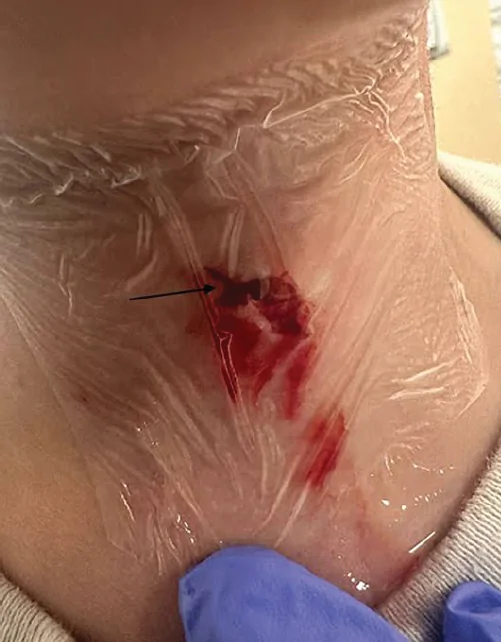
Medial anterior neck laceration (arrow) with overlying lidocaine-epinephrine-tetracaine gel and transparent, adhesive film dressing.

During the exam, he was breathing spontaneously with clear breath sounds and spoke in full sentences. He had sustained a 2–3 cm stellate laceration on the medial anterior neck, without active bleeding or visible foreign bodies. Additionally, he was found to have a 5-mm laceration to the second digit of the left hand, also without active bleeding. Lidocaine-epinephrine-tetracaine gel was applied by nursing staff for local anesthesia in the event the patient would require a laceration repair (Image 1).

While obtaining further history, the patient developed hoarseness, which his parents attributed to prolonged screaming and crying during the accident. Upon wound exploration in the ED, gentle probing revealed a track extending into the neck. Given the presence of hoarseness and the exam finding suggestive of a deeper track, the decision was made to proceed with CT of the neck. The imaging revealed a cluster of three hypodense foreign bodies lodged between the right lateral tracheal wall and the right common carotid artery just posterior to the lower pole of the right thyroid bone. The largest fragment measured 8 x 3 mm, with its tip lodged in the prevertebral muscles and abutting the tracheal wall. It was uncertain whether the foreign body had caused superficial injury to the tracheal wall, and there was no surrounding soft tissue emphysema to suggest tracheal perforation.

**Image 2:**
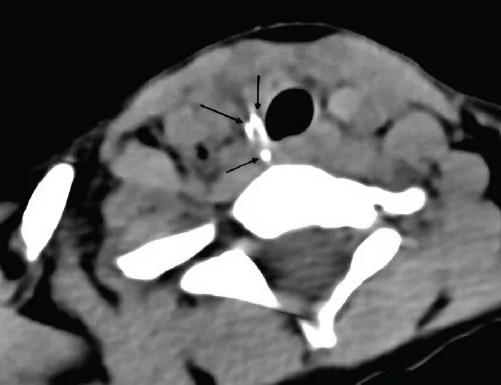
Axial view of a noncontrast computed tomography depicting glass foreign bodies in the neck of a 3-year-old male (arrows).

**Image 3:**
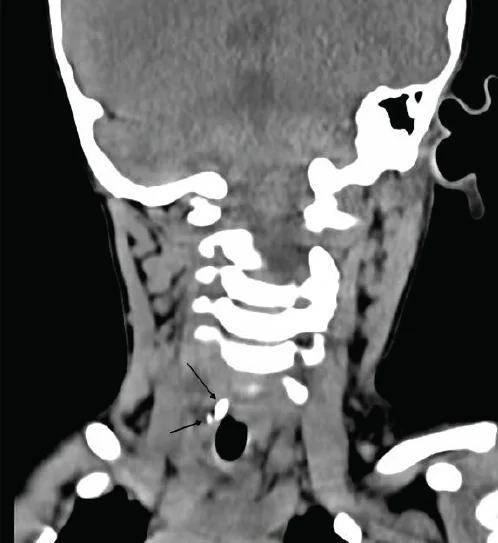
Coronal view of a non-contrast computed tomography imaging depicting glass foreign bodies (arrows).

The foreign body was also adjacent to the right lateral esophageal wall, but it did not appear to have penetrated the esophagus. Additionally, a 4 x 1 mm fragment was identified medial to the right common carotid artery, along with a 3 x 1 mm fragment situated just deep to the largest foreign body (Images 2 and 3). Trauma surgery was consulted, and the decision was made to take the patient to the operating room for foreign body removal and to rule out damage to critical cervical structures.

In the operating room, fluoroscopy failed to visualize the foreign bodies, complicating the open exploration. During exploration, the glass fragments were removed, and both esophagogastroduodenoscopy and laryngoscopy demonstrated no damage to the trachea or esophagus. The patient was admitted to the hospital and placed on cefazolin for 24 hours. He was then discharged with amoxicillin-clavulanate therapy and surgery follow-up. During follow-up visits, the wound was noted to be healing well with no erythema, discharge, or systemic symptoms such as fevers. He was cleared from surgical care at the two-week visit.

*CPC-EM Capsule*

What do we already know about this clinical entity?

*Penetrating neck injury in a child is a rare but dangerous diagnosis, as vital structures lie close beneath the skin of the neck.*

What makes this presentation of disease reportable?

*A superficial-appearing pediatric neck laceration concealed multiple glass foreign bodies adjacent to vascular and aerodigestive structures.*

What is the major learning point?

*Seemingly minor pediatric neck lacerations, especially from glass, warrant careful evaluation for penetrating neck injury and retained foreign bodies.*

How might this improve emergency medicine practice?

*This case highlights the need to maintain suspicion for penetrating neck injury in children and to evaluate with computed tomographyf in stable patients with soft signs of injury.*

On telephone follow-up four months following the initial presentation to the ED, the patient’s father stated that his son had recovered well. He conveyed appreciation for the care provided and consented to the publication of this case report.

## DISCUSSION

Penetrating trauma to the neck poses a serious risk due to the density of critical vascular, airway, and digestive structures in this area. Even seemingly minor injuries may conceal major damage to these internal structures, underscoring the need for a high index of suspicion to ensure timely diagnosis and management.^2^

Penetrating neck injury is defined as a wound that extends through the platysma.^3^ Most cases are the result of stab or gunshot wounds, but they may also be due to penetrating debris, including glass. Penetrating neck injury is more common in adult patients, with an incidence of only 0.28% in children experiencing trauma, as per a 2016 study using the National Trauma Data Bank.^4^ The low incidence of penetrating neck injury in children makes this topic hard to study. Due to their smaller anatomy and the proximity of the vital structures in their necks, penetrating neck injury in children can have severe consequences.^5^,^6^ Among pediatric patients, injuries to the aerodigestive tract are most common. Reported mortality from this injury in this population is 5.6%, with vascular injury representing the most common cause.^4^

Penetrating neck trauma management was previously determined based on an approach focused on anatomic zones of injury, initially described by Monson in 1969: zone 1 extending from the clavicles to the cricoid cartilage; zone 2 from the cricoid cartilage to the angle of the mandible; and zone 3 from the angle of the mandible to the base of the skull.^7^ However, reliance on the zonal approach led to unnecessary explorations, and the external wound location did not always correlate with the location of the internal injury. Current guidelines advocate for a “no zones” approach. Decision-making regarding imaging and wound exploration should be guided by physical examination findings, including both hard and soft signs of penetrating neck injury.^8^

Hard signs of penetrating neck injury include airway compromise, active hemorrhage, a rapidly expanding or pulsatile hematoma, shock, decreased or absent pulses, neurologic deficits, massive hemoptysis or hematemesis, massive subcutaneous emphysema, or a bubbling wound. Unstable patients or those with hard signs of injury require immediate surgical exploration, while stable patients with soft signs of injury are best evaluated with imaging, preferably CT with angiography. Soft signs of injury include dysphonia, hoarseness, a nonexpanding hematoma, mild hemoptysis, hematemesis, or subcutaneous emphysema.^3^,^7^,^9^–^11^ This approach is similar for both adult and pediatric patients.

Wounds caused by glass must be evaluated for the presence of foreign bodies due to its tendency to fragment. Wounds in areas not covered by clothing, such as the head, neck, and hands, carry a higher risk of foreign body retention. The presence of foreign bodies may initially be asymptomatic; therefore, a high index of suspicion is necessary to discover them. If missed in the neck, they can cause injury to vital cervical structures as well as complications such as persistent pain, delayed wound healing, deep neck space infection, fistula formation, or foreign body migration. Detection is often possible with radiologic evaluation, since most types of glass are radiopaque and visible on radiographs. However, detection may be limited by factors such as the surrounding tissue, fragment size, and glass composition. Computed tomography is considered the gold standard, providing accurate localization, reliable detection of all glass types, guidance for surgical planning, and identification of injuries to underlying structures.^1^

## CONCLUSION

This case highlights the importance of having a high index of suspicion for penetrating neck injury in children presenting with neck lacerations, particularly those caused by glass, given its tendency to fragment into penetrating debris. While rare in children, penetrating neck injury—even if seemingly minor—can be associated with devastating injury to the vital structures of the neck. Stable patients with soft signs of injury require further evaluation with CT imaging. Initial exploration in the ED at the bedside did not reveal any fragments in the wound, but the presence of a track beyond the platysma during gentle probing raised suspicion for a deeper penetrating foreign body.

The patient’s presentation with new hoarseness in his voice also raised the concern for airway involvement or a developing hematoma, prompting imaging. Had a CT not been performed, a radiograph would have been used to assess for retained glass fragments, as glass is usually radiopaque. However, given the failure of intraoperative fluoroscopy to demonstrate the foreign bodies, it is unclear whether a radiograph would have successfully identified the fragments. Therefore, in cases where clinical suspicion for a glass foreign body remains high despite negative radiographs, additional diagnostic evaluation is warranted.

## References

[1] Voss JO, Maier C, Wüster J (2021). Imaging foreign bodies in head and neck trauma: a pictorial review. Insights Imaging.

[2] Waseem M, Gernsheimer J (2010). A penetrating neck injury: trivial trauma with serious consequences. Pediatr Emerg Care.

[3] Alao T, Waseem M (2023). Neck trauma. StatPearls [Internet].

[4] Stone ME, Farber BA, Olorunfemi O (2016). Penetrating neck trauma in children: an uncommon entity described using the National Trauma Data Bank. J Trauma Acute Care Surg.

[5] Abdelmasih M, Kayssi A, Roche-Nagle G (2019). Penetrating paediatric neck trauma. BMJ Case Rep.

[6] Bustami RJ, Moses A, Imam AS (2019). No. 2 in zone 2: a case report of penetrating neck trauma in a child. Trauma Surg Acute Care Open.

[7] Shilston J, Evans DL, Simons A (2021). Initial management of blunt and penetrating neck trauma. BJA Educ.

[8] Ibraheem K, Khan M, Rhee P (2018). “No zone” approach in penetrating neck trauma reduces unnecessary computed tomography angiography and negative explorations. J Surg Res.

[9] Burgess CA, Dale OT, Almeyda R (2012). An evidence-based review of the assessment and management of penetrating neck trauma. Clin Otolaryngol.

[10] Nowicki JL, Stew B, Ooi E (2018). Penetrating neck injuries: a guide to evaluation and management. Ann R Coll Surg Engl.

[11] Tessler RA, Nguyen H, Newton C (2017). Pediatric penetrating neck trauma: hard signs of injury and selective neck exploration. J Trauma Acute Care Surg.

